# Hepatitis C virus genetic diversity by geographic region within genotype 1-6 subtypes among patients treated with glecaprevir and pibrentasvir

**DOI:** 10.1371/journal.pone.0205186

**Published:** 2018-10-04

**Authors:** Gretja Schnell, Preethi Krishnan, Rakesh Tripathi, Jill Beyer, Thomas Reisch, Michelle Irvin, Tatyana Dekhtyar, Liangjun Lu, Teresa I. Ng, Wangang Xie, Tami Pilot-Matias, Christine Collins

**Affiliations:** Research & Development, AbbVie Inc., North Chicago, Illinois, United States of America; University of Cincinnati College of Medicine, UNITED STATES

## Abstract

Hepatitis C virus (HCV) is genetically diverse and includes 7 genotypes and 67 confirmed subtypes, and the global distribution of each HCV genotype (GT) varies by geographic region. In this report, we utilized a large dataset of NS3/4A and NS5A sequences isolated from 2348 HCV GT1-6-infected patients treated with the regimen containing glecaprevir/pibrentasvir (GLE/PIB) to assess genetic diversity within HCV subtypes by geographic region using phylogenetic analyses, and evaluated the prevalence of baseline amino acid polymorphisms in NS3 and NS5A by region/country and phylogenetic cluster. Among 2348 NS3/4A and NS5A sequences, phylogenetic analysis identified 6 genotypes and 44 subtypes, including 3 GT1, 8 GT2, 3 GT3, 13 GT4, 1 GT5, and 16 GT6 subtypes. Phylogenetic analysis of HCV subtype 1a confirmed the presence of two clades, which differed by geographic region distribution and NS3 Q80K prevalence. We detected phylogenetic clustering by country in HCV subtypes 1a, 1b, 2a, 2b, and 5a, suggesting that genetically distinct virus lineages are circulating in different countries. In addition, two clades were detected in HCV GT4a and GT6e, and NS5A amino acid polymorphisms were differentially distributed between the 2 clades in each subtype. The prevalence of NS3 and NS5A baseline polymorphisms varied substantially by genotype and subtype; therefore, we also determined the activity of GLE or PIB against replicons containing NS3/4A or NS5A from HCV GT1-6 clinical samples representing 6 genotypes and 21 subtypes overall. GLE and PIB retained activity against the majority of HCV replicons containing NS3/4A or NS5A from HCV GT1-6 clinical samples, with a median EC_50_ of 0.29 nM for GLE and 1.1 pM for PIB in a transient replicon assay. The data presented in this report expands the available data on HCV epidemiology, subtype diversity by geographic region, and NS3 and NS5A baseline polymorphism prevalence.

## Introduction

Hepatitis C virus (HCV) infection affects 71 million people worldwide and is associated with liver disease and hepatocellular carcinoma [1–4]. HCV is genetically diverse and is classified into 7 genotypes, 67 confirmed subtypes, and 20 provisionally assigned subtypes [5]. The global distribution and prevalence of each HCV genotype (GT) varies by geographic region. HCV GT1 is the most prevalent worldwide and has a widespread geographic distribution, representing 46% of all HCV infections [1]. HCV GT3 is the second most prevalent genotype and accounts for 30% of global infections, and is more common in South Asia, Australasia, and some countries in Europe [1, 6]. HCV genotypes 2 and 4 are the next most common, each representing 9–13% of HCV infections with more limited geographic distribution. GT2 prevalence is higher in Asia and West Africa, while a high incidence of GT4 infection occurs in Central and Eastern sub-Saharan Africa, North Africa, and the Middle East [1, 6]. HCV genotypes 5, 6, and 7 are the most restricted in geographical distribution, with GT5 common in South Africa and GT6 prevalent in East and Southeast Asia [1], while GT7 infection has been reported in a small number of individuals from the Democratic Republic of Congo [7].

HCV genotypes and subtypes differ at the nucleotide level by approximately 30% and 15%, respectively, and subtype diversity varies by genotype [5]. HCV genotypes 2, 4, and 6 are the most diverse and include 11, 17, and 24 subtypes, respectively [5]. Subtype prevalence and diversity has been reported to vary by geographic region and country [6, 8]. In GT1a, two viral clades differ by geographical distribution and occurrence of nonstructural protein 3 (NS3) protease polymorphisms [9, 10]. Sequence clustering by geographic region has also been reported for other HCV subtypes based on phylogenetic analysis [8], indicating that genetically distinct virus lineages are circulating in different countries. Due to the high genetic diversity across HCV genomes, the efficacy of direct-acting antiviral (DAA) regimens for the treatment of HCV infection can be impacted by HCV genotype, subtype, and the presence of baseline amino acid polymorphisms [11]. Depending on the local prevalence of resistance-associated amino acid polymorphisms, the genetic diversity of HCV subtypes in different parts of the world may impact the treatment options available to HCV-infected patients.

Interferon-free HCV treatment options with DAAs have expanded from the earlier regimens of sofosbuvir (SOF) plus ribavirin (RBV) [12, 13], SOF plus daclatasvir [14], SOF plus simeprevir [14, 15], SOF/ledipasvir [16–18], ombitasvir/paritaprevir/ritonavir (OBV/PTV/r) with or without dasabuvir [19, 20], and elbasvir/grazoprevir [21, 22]. Newer HCV treatment options include pan-genotypic regimens with shorter treatment duration and indications for difficult-to-treat patient populations [23], including glecaprevir/pibrentasvir [24–30], SOF/velpatasvir (VEL) [31, 32], and SOF/VEL/voxilaprevir [33]. Glecaprevir (GLE) is an NS3/4A protease inhibitor (identified by AbbVie and Enanta) [34], and pibrentasvir (PIB) is an NS5A inhibitor [35]. The regimen of GLE/PIB is highly efficacious for the treatment of HCV GT1-6 infection [24–30]. Among 9 phase 2/3/3b studies that evaluated the safety and efficacy of GLE/PIB (300/120 mg dose) for the treatment of HCV GT1-6 infection, 2440 patients were enrolled in 27 countries that spanned five geographic regions worldwide, representing a large database of genetically and geographically diverse HCV GT1-6 patient samples. In this report, we utilized a large dataset of NS3/4A and NS5A sequences isolated from 2348 HCV GT1-6-infected patients treated with GLE/PIB to assess genetic diversity within HCV subtypes by geographic region, examine the prevalence of baseline amino acid polymorphisms by region/country and phylogenetic cluster, and determine the activity of GLE or PIB against subgenomic HCV replicons containing NS3/4A or NS5A from HCV GT1-6 clinical samples.

## Materials and methods

### Clinical studies

SURVEYOR-1 (ClinicalTrials.gov identifier NCT02243280) and SURVEYOR-2 (ClinicalTrials.gov identifier NCT02243293) were open label, dose-ranging, phase 2/3 studies that evaluated the safety, efficacy, and pharmacokinetics of GLE plus PIB with and without ribavirin (RBV) for 8, 12, or 16 weeks duration in 868 patients with chronic HCV GT1-6 infection, with or without compensated cirrhosis [27–29]. The HCV patient sequences analyzed from the SURVEYOR-1 and SURVEYOR-2 studies were isolated from the baseline samples of 590 patients who received the study dose of GLE 300 mg plus PIB 120 mg. The ENDURANCE-1, -2, -3, and -4 studies were phase 3 studies that evaluated the safety and efficacy of GLE/PIB (300/120 mg) for 8 or 12 weeks duration in 703, 302, 390, and 121 patients with chronic HCV infection without cirrhosis for GT1 (ENDURANCE-1, ClinicalTrials.gov identifier NCT02604017), GT2 (ENDURANCE-2, ClinicalTrials.gov identifier NCT02640482), GT3 (ENDURANCE-3, ClinicalTrials.gov identifier NCT02640157), and GT4, 5, or 6 (ENDURANCE-4, ClinicalTrials.gov identifier NCT02636595) [24, 30], respectively. ENDURANCE-1 included 670 patients with chronic HCV GT1 mono-infection and 33 patients with HIV-1/HCV GT1 co-infection. ENDURANCE-5/6 was a phase 3b, open-label study that evaluated the safety and efficacy of GLE/PIB (300/120 mg) for 8 or 12 weeks duration in 84 patients with chronic HCV GT5 or 6 infection, with or without compensated cirrhosis (ClinicalTrials.gov identifier NCT02966795) [36]. EXPEDITION-1 (ClinicalTrials.gov identifier NCT02642432) and EXPEDITION-4 (ClinicalTrials.gov identifier NCT02651194) were single arm, open-label, phase 3 studies that evaluated the safety and efficacy of GLE/PIB (300/120 mg) for 12 weeks duration in either 146 patients with chronic HCV GT 1, 2, 4, 5, or 6 infection and compensated cirrhosis (EXPEDITION-1) [25], or 104 patients with chronic severe renal impairment and chronic HCV GT1-6 infection with or without compensated cirrhosis (EXPEDITION-4) [26]. Clinical studies SURVEYOR-1, -2, ENDURANCE-1, -2, -3, -4, -5/6, and EXPEDITION-1, -4 enrolled HCV-infected patients that were treatment-naïve and those treatment-experienced to pegylated-interferon ± RBV ± SOF.

The current report is a post-hoc analysis of data from previously published multinational clinical trials SURVEYOR-1, -2, ENDURANCE-1, -2, -3, -4, -5/6, and EXPEDITION-1, -4. All clinical studies were conducted in accordance with the World Medical Association Declaration of Helsinki and the guidelines of the International Conference of Harmonization. The study protocols were approved by the relevant institutional review boards and regulatory agencies at each individual site, and all patients provided written informed consent. The names of the ethics committees and institutional review boards that approved these studies can be found on the trial registration at www.clinicaltrials.gov.

### Country of enrollment

HCV-infected patients from 27 countries were enrolled in clinical studies SURVEYOR-1, -2, ENDURANCE-1, -2, -3, -4, -5/6, and EXPEDITION-1, -4. A listing of country of enrollment by ISO country code is provided in S1 Table. Countries were grouped into the following geographic regions for analysis: Europe included the countries of AUT, BEL, CHE, DEU, ESP, FRA, GBR, GRC, HUN, ITA, LTU, POL, PRT, ROU, and SWE; North America included the countries of USA, CAN, MEX, and PRI; Asia included the countries of KOR, SGP (GT6 only), and TWN; Oceania included the countries of AUS and NZL; ROW (rest-of-world) included the countries of CHL (GT1), ISR (GT1), and ZAF (GT5a, GT6a).

### Subtype determination

Viral RNA isolation, reverse transcriptase (RT)-PCR, and nested PCR were conducted using genotype and subtype-specific primers on >2300 available baseline plasma samples, as previously described in detail for full-length NS3/4A and NS5A genes [34, 35, 37–41]. For each sample, HCV genotype was identified by the Versant HCV Genotype Inno-LiPA Assay v2.0 at the time of enrollment in the study, and HCV subtype was subsequently determined by neighbor-joining phylogenetic analysis of full-length NS3/4A and NS5A nucleotide sequences, as described in detail previously [40, 41]. The subtype for each sample was assigned based on agreement between NS3/4A and NS5A results when both gene sequences were available, or was based on data from a single gene target when data was not available from both genes.

### Next-generation sequencing

NGS analysis of NS3/4A and NS5A amplicons from 2348 baseline samples was conducted by DDL Diagnostic Laboratory (Rijswijk, Netherlands) for studies SURVEYOR-1, -2, ENDURANCE-1, -3, -4, -5/6, and EXPEDITION-1, and by Monogram Biosciences (South San Francisco, CA, USA) for studies ENDURANCE-2 and EXPEDITION-4, using methods that were described in detail previously [40]. PCR amplicons were purified and quantified by DDL Diagnostic Laboratory using methods previously described [40]. PCR amplicons were purified, quantified, and normalized by Monogram Biosciences using the AxyPrep Mag PCR Normalizer kit (Corning Life Sciences, Corning, NY). PCR amplicons were fragmented and tagged using the Nextera XT sample preparation and index kits (Illumina, San Diego, CA). Paired-end sequencing was conducted using the Illumina MiSeq platform, and FASTQ files were mapped against a subtype-specific reference sequence. Sequences were trimmed to remove nucleotides with a Q-score <25, and sequence reads less than 50 bases in length were discarded. Amino acid substitutions relative to a subtype-specific HCV reference sequence were reported by the sequencing vendor using a frequency threshold of ≥1%.

### Phylogenetic analysis

NGS consensus nucleotide sequences for NS3/4A and NS5A were generated with an ambiguity setting of 0.25 and aligned using MAFFT [42]. NS3/4A and NS5A sequences were grouped by genotype and subtype, and included in phylogenetic analyses to assess genetic relationships within each subtype by geographic region and country. Maximum likelihood phylogenetic trees were constructed using PHYML [43, 44] in Geneious software (Biomatters Ltd., Auckland, New Zealand) [45] with the HKY85 nucleotide substitution model [46], 100 bootstrapping replicates, and additional parameters as described in detail previously [40]. Phylogenetic analysis was conducted for both NS3/4A and NS5A for each subtype, and one target was selected as the representative tree displayed in figures. Sequence clusters that matched between the phylogenetic trees of NS3/4A and NS5A with bootstrap support ≥50 that contained ≥5 sequences were identified with a number, starting with C1 for the cluster with the greatest number of sequences.

### NS3 and NS5A baseline polymorphism analysis

NS3 amino acid positions 36, 43, 54, 55, 56, 80, 155, 156, 166 (GT3 only), and 168 were considered signature positions for the NS3/4A protease inhibitor class in GT1-6. Baseline amino acid polymorphisms were identified by comparing translated baseline sequences to the respective subtype-specific reference sequence shown in S2 Table. The number of patients with “Any” baseline polymorphism in NS3 at signature amino acid positions was also calculated. The prevalence of baseline polymorphisms in NS3 at a detection threshold ≥15% was analyzed by subtype, geographic region, and phylogenetic cluster. NS5A amino acid positions 24, 28, 30, 31, 32, 58, 92 and 93 were considered signature positions for the NS5A inhibitor class in GT1-6, and polymorphisms were identified by comparing translated baseline sequences to the respective subtype-specific reference sequence shown in S3 Table. The number of patients with “Any” baseline polymorphism in NS5A at signature amino acid positions was also calculated. The prevalence of baseline polymorphisms in NS5A at a detection threshold ≥15% was analyzed by subtype, geographic region, and phylogenetic cluster.

### HCV transient replicons containing NS3 or NS5A from GT1-6 clinical samples

The generation of HCV replicons containing NS3 or NS3/4A from subtype 1a, 1b, 2a, 2b, 3a, 4a, 4d, and 5a clinical samples for use in a transient transfection assay was previously described [34, 38]. For HCV subtypes 2c, 2i, 2l, 4f, 4g, 4k, 4o, and 4r, a consensus sequence for NS3/4A was derived from an alignment of 75, 7, 5, 3, 3, 8, 4, and 8 patient sequences, respectively. The NS3 consensus sequence for subtypes 2c, 2i, and 2l encompassed amino acids 1 to 225 of NS3, and was generated as a synthetic gene (IDT, Coralville, IA) and ligated into an HCV GT2a JFH1 (GenBank accession number AB047639) subgenomic transient replicon vector containing a luciferase reporter gene [47], in place of the corresponding region from 2a-JFH1. The NS3 consensus sequence for subtypes 4f, 4g, 4k, 4o, and 4r encompassed amino acids 1 to 251 of NS3, and was generated as a synthetic gene (IDT) and ligated into an HCV GT1b Con1 (GenBank accession number AJ238799) subgenomic transient replicon vector containing a luciferase reporter gene, in place of the corresponding region from 1b-Con1. For HCV GT6e, a consensus sequence for NS3/4A was derived from an alignment of 4 GT6e patient sequences. The full-length NS3/4A GT6e consensus sequence was generated as a synthetic gene (IDT) and ligated into the HCV GT1b Con1 transient replicon vector, in place of the corresponding region from 1b-Con1.

The generation of HCV replicons containing NS5A from subtype 1a, 1b, 2a, 2b, 3a, 4a, 4b, 4d, 4f, 4g, 4k, 4o, 4r, 5a, and 6a clinical samples for use in a transient transfection assay was previously described [37, 40, 41, 47]. For HCV subtypes 2c, 2i, 2l, 6e, and 6p, the NS5A gene encompassing amino acids 1 to 214 was amplified from individual clinical samples using subtype-specific PCR primers and ligated into an HCV GT1b Con1 subgenomic transient replicon vector containing a luciferase reporter gene [37], in place of the corresponding region from 1b-Con1. For HCV GT3b, a consensus sequence for NS5A was derived from an alignment of 10 GT3b patient sequences and the 3b-HCV-Tr sequence (GenBank accession number D49374). The NS5A GT3b consensus sequence encompassing amino acids 1 to 187 was generated as a synthetic gene (IDT) and ligated into an HCV GT2a JFH1 subgenomic transient replicon vector containing a luciferase reporter gene [47], in place of the corresponding region from 2a-JFH1.

The activity of GLE or PIB against chimeric transient replicons containing NS3, NS3/4A, or NS5A from GT1-6 clinical samples was assessed using the transient replicon assay as described in detail previously [34, 35, 37, 38, 40, 47]. The 50% effective concentration value (EC_50_) of GLE or PIB was calculated in Prism5 software (GraphPad Software, Inc., La Jolla, CA) using a nonlinear regression curve fitting to the 4-parameter logistic equation. The average EC_50_ value for each clinical sample was calculated from at least 2 independent experiments each conducted in duplicate.

### Statistical analysis

Fisher’s exact test with a two-sided significance level of 0.05 was used to compare sequence distribution by geographic region and phylogenetic clade or cluster, as well as the prevalence of baseline polymorphisms in NS3 and NS5A by geographic region and phylogenetic clade or cluster, without multiplicity adjustment.

## Results

### HCV GT1-6 subtypes by country of enrollment

**Fig 1:**
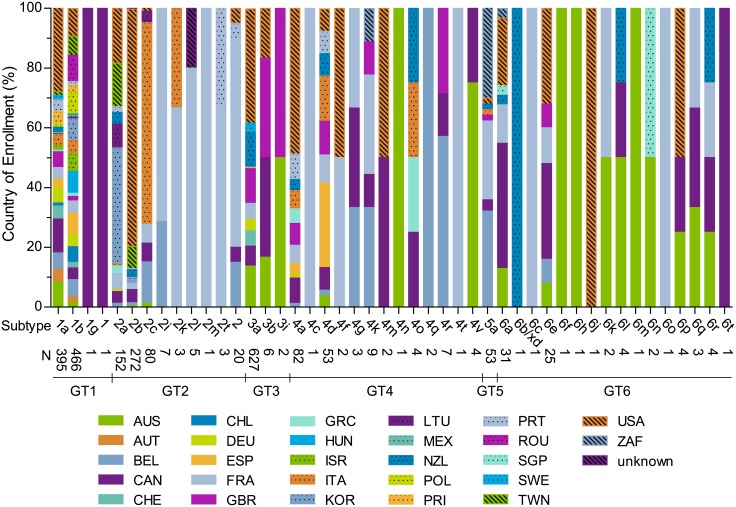
HCV GT1-6 subtypes by country of enrollment. The total number (N) of sequences and country (%) is listed for each subtype identified from 2348 available baseline samples. Countries are listed by ISO country code.

Among 2348 baseline samples, 44 HCV subtypes were identified by phylogenetic analysis of NS3/4A and NS5A sequences, including 3 GT1 (N = 863), 8 GT2 (N = 543), 3 GT3 (N = 635), 13 GT4 (N = 171), 1 GT5 (N = 53), and 16 GT6 (N = 83) subtypes (Fig 1). No GT7-infected patients were enrolled among the 9 phase 2/3/3b studies. Subtype diversity was highest in GT2, GT4, and GT6, and included 37 of the 52 confirmed subtypes among these 3 genotypes [5].

The distribution of countries was the most diverse for subtypes 1a and 1b, representing enrollment of patients from 22 and 24 countries, respectively. In subtype 1a (n = 395), 28.1% of patients were from the United States, 11.4% were from Canada, and 8.4% were from Australia. In subtype 1b (n = 466), the patient distribution was generally evenly divided among 24 countries, including 9.2%, 8.8%, and 7.5% of patients from the United States, Romania, and Poland, respectively. Six samples subtyped as either GT1a (n = 4) or GT1b (n = 2) by phylogenetic analysis of NS3/4A and NS5A sequences were included in subsequent sequence analyses, and were later determined to be GT2/1 chimeras based on full-genome sequencing; one subtype 1b sample from Korea and four subtype 1a samples from the United States were 2b/1 chimeras, and one subtype 1b sample from the United States was a 2k/1b chimera.

Among GT2-infected patients, subtypes 2a, 2b and 2c were the most prevalent (92.8%), and the country distribution varied depending on the subtype (Fig 1). In GT2a (n = 152), the majority of patients were from Asian countries of South Korea (38.8%) and Taiwan (14.5%), while in GT2b (n = 272) 79.4% of patients were from the United States. GT2c-infected patients (n = 80) were mainly from Italy (67.5%) and Belgium (13.8%), while the majority of patients infected with other GT2 subtypes (n = 39) were from France. The majority of the GT3-infected patients enrolled had subtype 3a infection (98.7%). Among GT3a-infected patients (n = 627), the majority were from North America or Oceania, including 38.3% from the United States, 13.7% from Australia, and 11.6% from New Zealand, while patients infected with subtype 3b (n = 6) or 3i (n = 2) were from Australia, Canada, or the United Kingdom. The majority of the GT4-infected patients had subtype 4a or 4d infection (78.9%, Fig 1). Among 82 GT4a-infected patients, 48.8% were from the United States, while GT4d-infected patients (n = 53) were predominantly from Europe, and patients infected with other GT4 subtypes (n = 36) were generally from Belgium, France, or Canada. In GT5a (n = 53), 30.2% of patients were from South Africa, 32.1% were from Belgium, and 26.4% were from France. GT6-infected patients were predominantly from Canada, the United States, France, and Australia (Fig 1).

### Two clades in HCV subtype 1a differ by geographic region and NS3 Q80K prevalence

**Fig 2:**
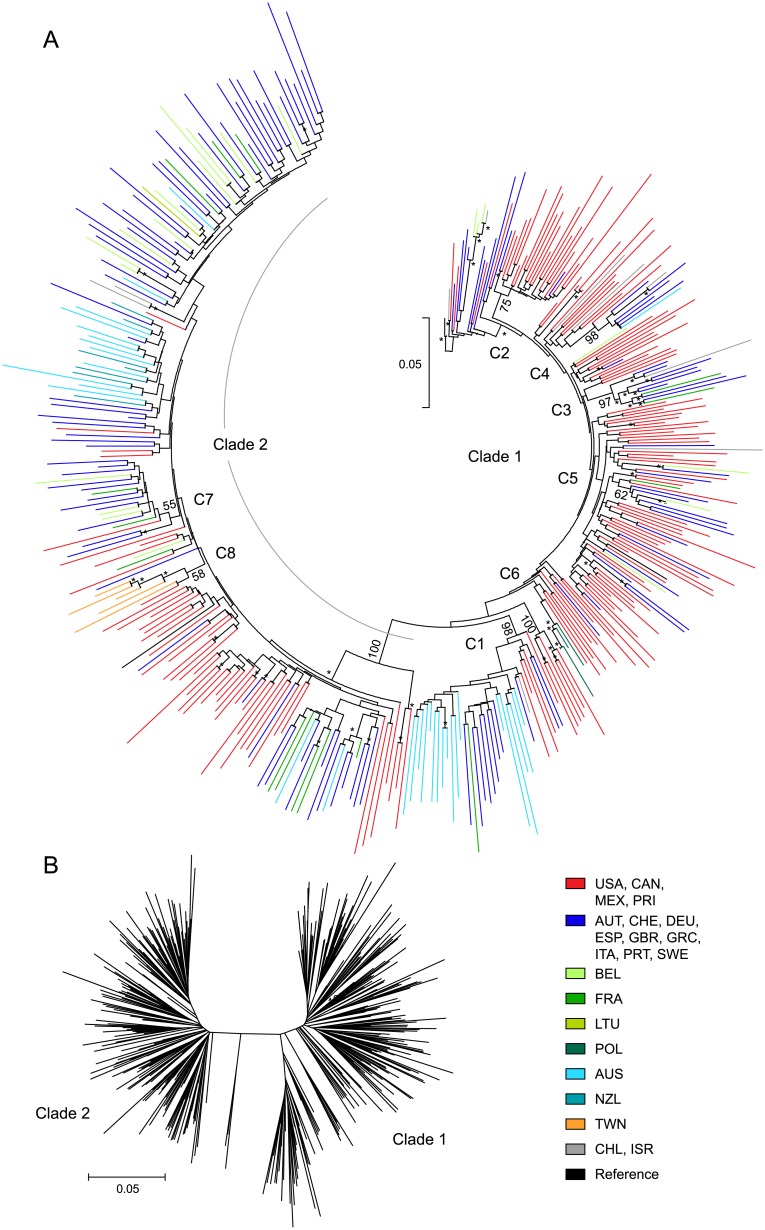
Two clades in HCV GT1a differ by geographic region distribution. Maximum likelihood phylogenetic tree displayed in (A) circular or (B) radial format for NS3/4A sequences from GT1a-infected patients. Bootstrap values are listed for nodes of sequence clustering, and bootstrap values ≥70 at other nodes in the tree are marked with an asterisk (*). Sequence clusters by geographic region were numbered starting at C1. The genetic distance scale bar indicates the number of nucleotide substitutions per site between sequences. HCV patient isolates are represented by color based on the country of enrollment.

Phylogenetic analysis of 395 NS3/4A and NS5A GT1a sequences confirmed the presence of 2 clades in HCV subtype 1a [10], and also identified 8 subgroups of sequence clusters in either clade 1 (C1-C6) or clade 2 (C7 and C8) (Fig 2). An analysis of geographic region based on phylogenetic clade classification revealed that the distribution of sequences from North America and Europe was significantly different between clade 1 and clade 2 (P-value <0.0001). The geographic region distribution of clade 1 (n = 215) was 56% North America, 32% Europe, 9% Oceania, and 4% ROW, while the distribution of clade 2 (n = 180) was 30% North America, 55% Europe, 3% Asia, 12% Oceania, and 1% ROW (Table 1).

**Table 1:** Country distribution by phylogenetic clade and cluster.

Subtype, Phylo. Cluster	n (%)^a^											N^a^
	North America	Europe					Asia		Oceania		ROW	
		EU-other^b^	BEL	FRA	LTU	POL	KOR	TWN	AUS	NZL		
**GT1a**^c^	**174 (44)**	**124 (31)**	**22 (6)**	**15 (4)**	**1 (0.3)**	**4 (1)**	**0**	**5 (1)**	**33 (8)**	**7 (2)**	**10 (3)**	**395**
Clade 1	120 (56)	52 (24)	8 (4)	4 (2)		4 (2)			19 (9)		8 (4)	215
Clade 2	54 (30)	72 (40)	14 (8)	11 (6)	1 (0.6)			5 (3)	14 (8)	7 (4)	2 (1)	180
C1	5 (14)	11 (31)		1 (3)					18 (50)		1 (3)	36
C2	22 (92)	2 (8)										24
C3		8 (73)		2 (18)							1 (9)	11
C4	1 (12)	5 (63)							1 (12)		1 (12)	8
C5		5 (71)	2 (29)									7
C6	6 (100)											6
C7	1 (6)	11 (65)	3 (18)	2 (12)								17
C8	1 (17)							5 (83)				6
**GT1b**	**74 (16)**	**182 (39)**	**27 (6)**	**19 (4)**	**3 (0.6)**	**35 (8)**	**34 (7)**	**30 (6)**	**8 (2)**	**2 (0.4)**	**52 (11)**	**466**
C1							2 (15)	11 (85)				13
C2	1 (8)	4 (31)	2 (15)	1 (8)		2 (15)					3 (23)	13
C3	10 (83)	2 (17)										12
C4	1 (10)	7 (70)		1 (10)							1 (10)	10
C5		1 (10)				9 (90)						10
C6						7 (100)						7
C7		1 (14)				1 (14)					5 (71)	7
C8							4 (80)	1 (20)				5
C9									5 (100)			5
**GT2a**	**34 (22)**	**9 (6)**	**2 (1)**	**8 (5)**	**12 (8)**	**0**	**59 (39)**	**22 (14)**	**0**	**6 (4)**	**0**	**152**
C1	8 (42)	4 (21)		7 (37)								19
C2	2 (11)	1 (6)	2 (11)	1 (6)	12 (67)							18
C3							7 (100)					7
C4	1 (17)									5 (83)		6
**GT2b**	**228 (84)**	**1 (0.4)**	**3 (1)**	**6 (2)**	**0**	**0**	**5 (2)**	**20 (7)**	**1 (0.4)**	**7 (3)**	**0**	**272**
C1	17 (100)											17
C2								11 (100)				11
C3							4 (40)	6 (60)				10
C4	10 (100)											10
C5	9 (100)											9
C6	8 (100)											8
C7										7 (100)		7
C8			2 (33)	4 (67)								6
**GT3a**	**282 (45)**	**152 (24)**	**0**	**34 (5)**	**0**	**0**	**0**	**0**	**86 (14)**	**73 (12)**	**0**	**627**
C1	2 (33)	4 (67)										6
**GT4a**	**47 (57)**	**26 (32)**	**1 (1)**	**5 (6)**	**0**	**0**	**0**	**0**	**0**	**3 (4)**	**0**	**82**
Clade 1	14 (40)	15 (43)	1 (3)	2 (6)						3 (9)		35
Clade 2	33 (70)	11 (23)		3 (6)								47
**GT5a**	**3 (6)**	**2 (4)**	**17 (32)**	**14 (26)**	**0**	**0**	**0**	**0**	**0**	**1 (2)**	**16 (30)**	**53**
C1			16 (100)									16
C2											6 (100)	6
C3				5 (100)								5
**GT6a**	**20 (65)**	**0**	**0**	**4 (13)**	**0**	**0**	**1**^d^ **(3)**		**4 (13)**	**1 (3)**	**1 (3)**	**31**
C1	4 (57)						1^d^ (14)			1 (14)	1 (14)	7
**GT6e**	**16 (64)**	**2 (8)**	**2 (8)**	**3 (12)**	**0**	**0**	**0**	**0**	**2 (8)**	**0**	**0**	**25**
Clade 1	7 (78)	1 (11)	1 (11)									9
Clade 2	9 (56)	1 (6)	1 (6)	3 (19)					2 (13)			16

^a^n, number of sequences per country; N, total number of available sequences; % = n/N

^b^EU-other includes the countries of AUT, CHE, DEU, ESP, GBR, GRC, HUN, ITA, PRT, ROU, and SWE.

^c^In GT1a, C1-C6 are subgroups of Clade 1; C7 and C8 are subgroups of Clade 2.

^d^In GT6a, the Asia sequence was from SGP.

Further assessment of sequences comprising clade 1 revealed that 62% (42/68) of sequences from European countries clustered independently from the majority of sequences from North America (90%; 108/120). In addition, 94.7% (18/19) of the clade 1 sequences from Australia sorted together in cluster C1. Among the 6 sequence clusters identified in clade 1, cluster C1 was the largest and included 36 sequences, of which 50% were from Australia, 33% were from Europe, and 14% were from North America (Table 1). Clusters C3, C4, C5, and C7 contained predominantly sequences from Europe (63–100%; Table 1). All of the GT1a sequences from Taiwan (5/5) clustered in C8 within clade 2.

The prevalence of baseline polymorphisms in GT1a for NS3 and NS5A was assessed by geographic region and phylogenetic clade, and is shown in Tables 2 and 3, respectively. Polymorphisms at NS3 amino acid position A156 were not detected in GT1-6 sequences. The prevalence of polymorphisms at NS3 amino acid positions 36, 54, 55, 56, 155, and 168 was similar across geographic region and phylogenetic clade (GT1a, Table 2). The prevalence of Q80K/L/M/R in NS3 was 37% overall (145/391) while Q80K alone was 35% (136/391), and the prevalence varied significantly depending on the geographic region and phylogenetic clade classification (Table 2). Based on geographic region, Q80K prevalence was significantly higher (P-value <0.0001) in sequences from North America (56%; 96/170) compared to Europe (21%, 35/166) or Oceania (2.5%; 1/40). The prevalence of Q80K was also significantly different between clade 1 (59%, 127/214) and clade 2 (5.1%, 9/177; P-value <0.0001). In addition, sequence clusters C1-C6 in clade 1 were generally grouped by the presence or absence of Q80K in NS3. Of the 84 sequences in clade 1 with Q80 in NS3, 67% (56/84) clustered in C1, C3, and C6, while clusters C2 (n = 24) and C4 (n = 8) each contained 100% sequences with Q80K.

**Table 2:** NS3 baseline polymorphism prevalence in GT1-6 by geographic region and phylogenetic cluster.

Subtype, Region, Phylo. Cluster	NS3 Amino Acid Polymorphisms, n (%)^a^								N^a^
	36	54	55	56	80	155	168	Any	
**GT1a**^b^	V36A/L/M	T54S	V55A/I/P	Y56F	Q80K/L/M/R	R155K	D168E		
All	13 (3)	8 (2)	19 (5)	1 (0.3)	145 (37)	4 (1)	4 (1)	174 (45)	391
Europe	5 (3)	4 (2)	5 (3)		40 (24)	1 (0.6)	3 (2)	51 (31)	166
North America	5 (3)	3 (2)	10 (6)	1 (0.6)	100 (59)	1 (0.6)	1 (0.6)	110 (65)	170
Asia	1 (20)							1 (20)	5
Oceania	1 (3)	1 (3)	4 (10)		1 (3)	1 (3)		6 (15)	40
ROW	1 (10)				4 (40)	1 (10)		5 (50)	10
Clade 1	7 (3)	5 (2)	13 (6)	1 (0.5)	130 (61)	2 (1)	1 (0.5)	143 (67)	214
Clade 2	6 (3)	3 (2)	6 (3)		15 (8)	2 (1)	3 (2)	31 (18)	177
C1	1 (3)	1 (3)	5 (14)		(0)			6 (17)	36
C2			2 (8)		24 (100)		1 (4)	24 (100)	24
C3	1 (9)				1 (9)	1 (9)		3 (27)	11
C4					8 (100)			8 (100)	8
C5	1 (14)	1 (14)	1 (14)		6 (86)			7 (100)	7
C6					1 (17)			1 (17)	6
C7					3 (18)			3 (18)	17
C8	1 (17)							1 (17)	6
**GT1b**	V36I/L	T54S	V55A/P	Y56F	Q80H/K/L/R	R155	D168E		
All	7 (2)	8 (2)	3 (0.7)	147 (32)	23 (5)	0	1 (0.2)	169 (37)	461
Europe	3 (1)	3 (1)	2 (0.8)	95 (36)	5 (2)			105 (40)	263
[Poland]^c^				18 (51)	2 (6)			19 (54)	35
[Spain]^c^				18 (53)				18 (53)	34
North America	1 (1)	1 (1)	1 (1)	17 (23)	4 (6)			21 (29)	73
Asia	3 (5)	3 (5)		16 (25)	11 (18)			22 (35)	63
Oceania					1 (10)			1 (10)	10
ROW		1 (2)		19 (37)	2 (4)		1 (2)	20 (39)	52
C1				1 (8)	2 (15)			2 (15)	13
C2		1 (8)		3 (23)	1 (8)			5 (39)	13
C3		1 (9)						1 (9)	11
C4				7 (70)	2 (20)			7 (70)	10
C5				6 (60)				6 (60)	10
C6				2 (29)				2 (29)	7
C7				7 (100)	2 (29)			7 (100)	7
C8								0	5
C9								0	5
**GT1-other**^d^	V36L	T54S	V55	Y56	Q80	R155	D168		
All	1 (50)	1 (50)						2 (100)	2
**GT2a**	L36M	T54A	V55	Y56F	G80	R155	D168E		
All	3 (2)	1 (0.7)	0	10 (7)	0	0	3 (2)	16 (11)	144
Europe	1 (3)			1 (3)			1 (3)	2 (7)	29
North America	2 (6)			2 (6)			1 (3)	5 (15)	34
Asia		1 (1)		7 (9)			1 (1)	9 (12)	75
Oceania								0	6
C1								0	19
C2	1 (6)			1 (6)				1 (6)	16
C3				6 (86)				6 (86)	7
C4								0	6
**GT2b**	L36	T54	V55I	Y56F	G80	R155	D168V		
All	0	0	1 (0.4)	44 (17)	0	0	1 (0.4)	45 (18)	254
Europe				1 (10)				1 (10)	10
North America			1 (0.5)	37 (18)			1 (0.5)	38 (18)	211
Asia				5 (21)				5 (21)	24
Oceania				1 (13)				1 (13)	8
C1				1 (8)				1 (8)	13
C2				2 (20)				2 (20)	10
C3				3 (30)				3 (30)	10
C4				1 (10)				1 (10)	10
C5				2 (25)				2 (25)	8
C6				1 (13)				1 (13)	8
C7				1 (14)				1 (14)	7
C8								0	6
**GT2c**	L36	T54	V55	F56	G80	R155	D168	0	71
**GT2-other**^e^	L36	T54	V55	Y56F	G80	R155	D168		
All	0	0	0	11 (65)	0	0	0	11 (65)	17
**GT3a**^f^	L36	T54A/S	V55I	Y56F	Q80K	R155	Q168K/R		
All	0	3 (0.5)	2 (0.3)	1 (0.2)	1 (0.2)	0	10 (2)	99^f^ (16)	624
Europe		1 (0.5)	1 (0.5)				3 (2)	34^f^ (18)	185
North America		1 (0.4)		1 (0.4)	1 (0.4)		7 (3)	41^f^ (15)	281
Oceania		1 (0.6)	1 (0.6)					24^f^ (15)	158
C1								1^f^ (17)	6
**GT3-other**^g^	L36	T54	V55	Y56	Q80	R155	Q168	0	8
**GT4a**	L36	T54S	V55	Y56	Q80	R155	D168		
All	0	4 (5)	0	0	0	0	0	4 (5)	81
Europe								0	31
North America		4 (9)						4 (9)	47
Oceania								0	3
Clade 1		2 (6)						2 (6)	34
Clade 2		2 (4)						2 (4)	47
**GT4d**	L36	T54	V55	Y56	Q80K	R155	D168E		
All	0	0	0	0	1 (2)	0	1 (2)	2 (4)	52
**GT4-other**^h^	L36	T54	V55	Y56F	Q80	R155	D168E		
All	0	0	0	1 (3)	0	0	2 (6)	3 (8)	36
**GT5a**	L36	T54	V55I	F56Y	K80	R155	D168E		
All	0	0	1 (2)	1 (2)	0	0	25 (47)	26 (49)	53
Europe			1 (3)	1 (3)			15 (46)	16 (49)	33
North America							1 (33)	1 (33)	3
Oceania							1 (100)	1 (100)	1
S. Africa							8 (50)	8 (50)	16
C1			1 (6)	1 (6)			7 (44)	8 (50)	16
C2							3 (50)	3 (50)	6
C3							3 (60)	3 (60)	5
**GT6a**	V36	T54	V55	Y56	L80K/R	R155	D168E		
All	0	0	0	0	30 (100)	0	2 (7)	30 (100)	30
Europe					4 (100)			4 (100)	4
North America					19 (100)		2 (11)	19 (100)	19
Asia					1 (100)			1 (100)	1
Oceania					5 (100)			5 (100)	5
S. Africa					1 (100)			1 (100)	1
C1					7 (100)			7 (100)	7
**GT6e**	V36L	T54	V55	Y56	Q80	R155	D168		
All	2 (8)	0	0	0	0	0	0	2 (8)	24
Europe								0	7
North America	1 (7)							1 (7)	15
Oceania	1 (50)							1 (50)	2
Clade 1								0	8
Clade 2	2 (13)							2 (13)	16
**GT6-other**^i^	V36I/L	T54	V55	Y56F	L80Q	R155	D168E		
All	7 (29)	0	0	5 (21)	24 (100)	0	1 (4)	24 (100)	24
Europe	2 (50)			1 (25)	4 (100)			4 (100)	4
North America	3 (43)			1 (14)	7 (100)			7 (100)	7
Asia					1 (100)			1 (100)	1
Oceania	2 (17)			3 (25)	12 (100)		1 (8)	12 (100)	12

^a^n = number of sequences per country; N, total number of available sequences; % = n/N.

^b^In GT1a, C1-C6 are subgroups of Clade 1; C7 and C8 are subgroups of Clade 2.

^c^Poland and Spain are included in the numbers for Europe.

^d^GT1-other included 1 GT1 and 1 GT1g sequence from North America. Polymorphisms were assessed using the 1b-Con1 reference sequence.

^e^GT2-other included 4 GT2, 6 GT2i, 1 GT2k, 4 GT2l, and 2 GT2t sequences; all sequences were from Europe. Polymorphisms were assessed using the 2b-HC-J8 reference sequence.

^f^In GT3a, “Any” count also includes polymorphisms at amino acid positions 43 and 166 in NS3. Polymorphisms A166S/T were detected in 32/185, 33/281, and 21/158 sequences from Europe, North America, and Oceania, respectively. F43L was detected in 1 sequence from Oceania. In phylogenetic cluster C1, A166S/T was detected in 1/6 sequences.

^g^GT3-other included 2 GT3i and 6 GT3b sequences. Polymorphisms were assessed using the 3a-S52 reference sequence.

^h^GT4-other included 1 GT4c, 2 GT4f, 3 GT4g, 9 GT4k, 2 GT4m, 1 GT4n, 4 GT4o, 2 GT4q, 7 GT4r, 1 GT4t, and 4 GT4v sequences. Polymorphisms were assessed using the 4a-ED43 reference sequence. D168E was detected in 1/22 and 1/8 sequences from Europe and North America, respectively. Y56F was detected in 1/8 sequences from North America.

^i^GT6-other included 1 GT6b/6xd, 1 GT6f, 1 GT6h, 1 GT6j, 2 GT6k, 3 GT6l, 1 GT6m, 2 GT6n, 1 GT6o, 4 GT6p, 3 GT6q, and 4 GT6r sequences. Polymorphisms were assessed using the 6a-EUHK2 reference sequence.

**Table 3:** NS5A baseline polymorphism prevalence in GT1-6 by geographic region and phylogenetic cluster.

Subtype, Region, Phylo. Cluster	NS5A Amino Acid Polymorphisms, n (%)^a^								N^a^
	24	28	30	31	58	92	93	Any	
**GT1a**^b^	K24Q/R	M28I/L/V/T	Q30H/L/R	L31M	H58D/L/P/Q/R/Y	A92P	Y93C/H/N		
All	6 (2)	33 (9)	7 (2)	8 (2)	25 (7)	1 (0.3)	8 (2)	78 (20)	387
Europe	3 (2)	18 (11)	3 (2)	2 (1)	12 (8)	1 (0.6)	2 (1)	35 (22)	161
North America	1 (0.6)	13 (8)	4 (2)	6 (4)	8 (5)		6 (4)	34 (20)	172
Asia	1 (20)	1 (20)						2 (40)	5
Oceania	1 (3)	1 (3)			5 (13)			7 (18)	39
ROW								0	10
Clade 1	3 (1)	19 (9)	2 (1)	6 (3)	12 (6)	1 (0.5)	5 (2)	42 (20)	212
Clade 2	3 (2)	14 (8)	5 (3)	2 (1)	13 (7)		3 (2)	36 (21)	175
C1	1 (3)	1 (3)			4 (11)			6 (17)	35
C2		1 (4)		1 (4)	2 (8)			4 (17)	24
C3	2 (20)							2 (20)	10
C4								0	8
C5		2 (29)						2 (29)	7
C6				1 (17)				1 (17)	6
C7							1 (6)	1 (6)	16
C8	1 (17)	1 (17)						2 (33)	6
**GT1b**	Q24K/R	L28M	R30K/L/M/Q	L31I/M	P58A/L/Q/R/S/T	A92E/T/V	Y93F/H/S		
All	5 (1)	11 (2)	22 (5)	21 (5)	44 (10)	35 (8)	38 (8)	147 (32)	462
Europe	2 (0.8)	6 (2)	12 (5)	12 (5)	23 (9)	16 (6)	20 (8)	75 (29)	263
North America	2 (3)	1 (1)	4 (5)	3 (4)	6 (8)	6 (8)	4 (5)	21 (28)	74
Asia	1 (2)	2 (3)	1 (2)	2 (3)	12 (19)	8 (13)	8 (13)	29 (46)	63
Oceania				1 (10)		1 (10)	1 (10)	3 (30)	10
ROW		2 (4)	5 (10)	3 (6)	3 (6)	4 (8)	5 (10)	19 (37)	52
C1							3 (25)	3 (25)	12
C2		1 (8)					1 (8)	2 (15)	13
C3						4 (33)	1 (8)	5 (42)	12
C4			1 (10)		1 (10)	1 (10)		2 (20)	10
C5						1 (10)		1 (10)	10
C6							1 (14)	1 (14)	7
C7				1 (14)		1 (14)	2 (29)	3 (43)	7
C8						4 (80; A92T)		4 (80)	5
C9						1 (20)		1 (20)	5
**GT1-other**^c^	Q24K/R	L28	R30Q/T	L31M	P58	A92	Y93F/H		
All	2 (100)		2 (100)	1 (50)			2 (100)	2 (100)	2
**GT2a**	T24A/S	F28C/L/V	K30R	L31M/V	P58S	C92N/S	Y93		
All	15 (10)	6 (4)	2 (1)	141 (94)	4 (3)	7 (5)	0	141 (94)	150
Europe	3 (10)		1 (3)	31 (100)		3 (10)		31 (100)	31
North America	3 (9)	1 (3)	1 (3)	33 (97)	1 (3)	1 (3)		33 (97)	34
Asia	5 (6)	4 (5)		71 (90)	3 (4)	3 (4)		71 (90)	79
Oceania	4 (67)	1 (17)		6 (100)				6 (100)	6
C1			1 (5)	19 (100)		3 (16)		19 (100)	19
C2				18 (100)	1 (6)	1 (6)		18 (100)	18
C3				5 (83)				5 (83)	6
C4	4 (67)	1 (17)		6 (100)				6 (100)	6
**GT2b**	S24	L28F	K30R	M31I/L/V	P58A/S	C92S	Y93		
All	0	8 (3)	1 (0.4)	182 (69)	12 (5)	3 (1)	0	191 (72)	265
Europe				8 (89)				8 (89)	9
North America		6 (3)		152^d^ (68)	10 (5)	2 (1)		158 (71)	223
Asia		2 (8)	1 (4)	13 (54)	2 (8)	1 (4)		16 (67)	24
Oceania				8 (100)				8 (100)	8
C1		1 (6)		3 (18; M31I/V)				4 (24)	17
C2			1 (9)	8 (73)	2 (18)			9 (82)	11
C3		1 (11)		4 (44)				5 (56)	9
C4				6 (60)				6 (60)	10
C5		1 (11)				1 (11)		1 (11)	9
C6		1 (13)		4 (50)	1 (13)			5 (63)	8
C7				7 (100)				7 (100)	7
C8				6 (100)				6 (100)	6
**GT2c**^e^	S24A	F28C	R30K	L31F/M	P58A	C92S/W	Y93		
All	1 (1)	34 (43)	72 (90)	14 (18)	1 (1)	2 (3)	0	73 (91)	80
**GT2-other**^f^	S24T	L28C/F/M	K30R	M31L	P58S/T	C92S	Y93		
All	1 (3)	21 (55)	5 (13)	13 (34)	2 (5)	5 (13)	0	32 (91)	38
**GT3a**^g^	S24A	M28V	A30K/L/M/R/S/T/V^g^	L31	P58A/R/S/T	E92D/G	Y93H		
All	12 (2)	7 (1)	73 (12)	0	22 (4)	2 (0.3)	31 (5)	132 (21)	626
Europe	7 (4)	2 (1)	28 (15)		8 (4)		7 (4)	48 (26)	186
North America	5 (2)	3 (1)	24 (9)		6 (2)	1 (0.4)	11 (4)	47 (17)	281
Oceania		2 (1)	21 (13)		8 (5)	1 (0.6	13 (8)	37 (23)	159
C1			1 (17)					1 (17)	6
**GT3-other**^h^	S24	M28	A30K/M	L31M	P58	E92	Y93		
All	0	0	8 (100)	6 (75)	0	0	0	8 (100)	8
**GT4a**	K24	L28M	L30R	M31	P58L/S/T	A92	Y93		
All	0	9 (11)	8 (10)	0	5 (6)	0	0	20 (25)	81
Europe		3 (10)	4 (13)		1 (3)			8 (26)	31
North America		6 (13)	4 (9)		4 (9)			12 (26)	47
Oceania								0	3
Clade 1		3 (9)	6 (17)		2 (6)			10 (29)	35
Clade 2		6 (13)	2 (4)		3 (7)			10 (22)	46
**GT4d**^i^	K24	L28	R30	M31L/V	T58A/L/P	A92	Y93		
All	0	0	0	2 (4)	43 (83)	0	0	43 (83)	52
**GT4-other**^j^	K24	L28I/M/V	L30A/C/H/R/S/T	M31L	P58T	A92	Y93H		
All	0	11 (33)	33 (100)	18 (55)	2 (6)	0	1 (3)	33 (100)	33
**GT5a**	Q24	L28	Q30L/R	L31F	P58S	A92S	T93		
All	0	0	3 (6)	1 (2)	1 (2)	1 (2)	0	6 (11)	53
Europe			3 (9)					3 (9)	33
North America								0	3
Oceania								0	1
South Africa				1 (6)	1 (6)	1 (6)		3 (19)	16
C1								0	16
C2				1 (17)	1 (17)			2 (33)	6
C3			1 (20)					1 (20)	5
**GT6a**	Q24K/R	F28L	R30	L31M	T58	A92	T93		
All	5 (16)	22 (71)	0	1 (3)	0	0	0	23 (74)	31
Europe		3 (75)						3 (75)	4
North America	4 (20)	13 (65)		1 (5)				13 (65)	20
Asia		1 (100)						1 (100)	1
Oceania	1 (20)	4 (80)						5 (100)	5
South Africa		1 (100)						1 (100)	1
C1	1 (14)	5 (71)						5 (71)	7
**GT6e**	K24R	V28M	S30	L31I	P58S	A92	T93S		
All	5 (20)	10 (40)	0	1 (4)	3 (12)	0	2 (8)	13 (52)	25
Europe	1 (14)	2 (29)			1 (14)		1 (14)	3 (43)	7
North America	4 (25)	8 (50)		1 (6)	2 (13)		1 (6)	10 (63)	16
Oceania								0	2
Clade 1	5 (56)	8 (89)						8 (89)	9
Clade 2		2 (13)		1 (6)	3 (19)		2 (13)	5 (31)	16
**GT6-other**^k^	Q24K/R	F28A/LM/T/V/Y	R30A/S/T/V	L31I/M	T58A/G/P/S	A92	T93S		
All	27 (100)	27 (100)	26 (96)	1 (4)	24 (90)	0	6 (22)	27 (100)	27
Europe	5 (100)	5 (100)	5 (100)		5 (100)		1 (20)	5 (100)	5
North America	8 (100)	8 (100)	8 (100)		8 (100)		2 (25)	8 (100)	8
Asia	1 (100)	1 (100)	1 (100)				1 (100)	1 (100)	1
Oceania	13 (100)	13 (100)	12 (92)	1 (7)	11 (85)		2 (15)	13 (100)	13

^a^n = number of sequences per country; N, total number of available sequences; % = n/N.

^b^In GT1a, C1-C6 are subgroups of Clade 1; C7 and C8 are subgroups of Clade 2.

^c^GT1-other category included 1 GT1 and 1 GT1g sequence from North America. Polymorphisms were assessed using the 1b-Con1 reference sequence.

^d^Includes 3 sequences with M31I/V and 149 sequences with M31L.

^e^In GT2c, 73 sequences were from Europe, 6 were from North America, and 1 was from Oceania.

^f^GT2-other category included 20 GT2, 7 GT2i, 3 GT2k, 5 GT2l, 1 GT2m, and 2 GT2t sequences; 35 sequences were from Europe and 2 sequences were from North America. Polymorphisms were assessed using the 2b-HC-J8 reference sequence.

^g^In GT3a, A30K prevalence was 9.7% (18/186) in Europe, 4.6% (13/281) in North America, and 5.0% (8/159) in Oceania. One sequence from Europe had A30K as well as Y93H in NS5A. One sequence in cluster C1 contained A30K.

^h^GT3-other category included 2 GT3i and 6 GT3b sequences. Polymorphisms were assessed using the 3a-S52 reference sequence.

^i^In GT4d, both sequences with the M31L/V polymorphism were from Europe. Polymorphisms at amino acid position 58 were detected in 31/39 (79%), 7/8 (88%), and 5/5 (100%) sequences from Europe, North America, and Oceania, respectively.

^j^GT4-other category included 1 GT4c, 2 GT4f, 2 GT4g, 8 GT4k, 2 GT4m, 1 GT4n, 3 GT4o, 2 GT4q, 7 GT4r, 1 GT4t, and 4 GT4v sequences. Polymorphisms were assessed using the 4a-ED43 reference sequence.

^k^GT6-other category included 1 GT6b/6xd, 1 GT6c, 1 GT6f, 1 GT6h, 1 GT6j, 2 GT6k, 4 GT6l, 1 GT6m, 2 GT6n, 1 GT6o, 4 GT6p, 3 GT6q, 4 GT6r, and 1 GT6t sequences. Polymorphisms were assessed using the 6a-EUHK2 reference sequence.

In NS5A, the prevalence of polymorphisms at amino acid positions 24, 30, 31, 92, and 93 was similar across geographic region and phylogenetic clade (GT1a, Table 3). Polymorphisms at NS5A amino acid position P32 were not detected in GT1-6 sequences. In the Oceania region, NS5A polymorphisms at position M28 were less prevalent (3%, 1/39), while polymorphisms at position H58 were more common (13%, 5/39) compared to other geographic regions. NS5A polymorphisms at positions important for the inhibitor-class had a similar prevalence between clade 1 and 2 in subtype 1a.

### Phylogenetic clustering by geographic region in HCV subtypes 1b, 2a, 2b, and 5a

**Fig 3:**
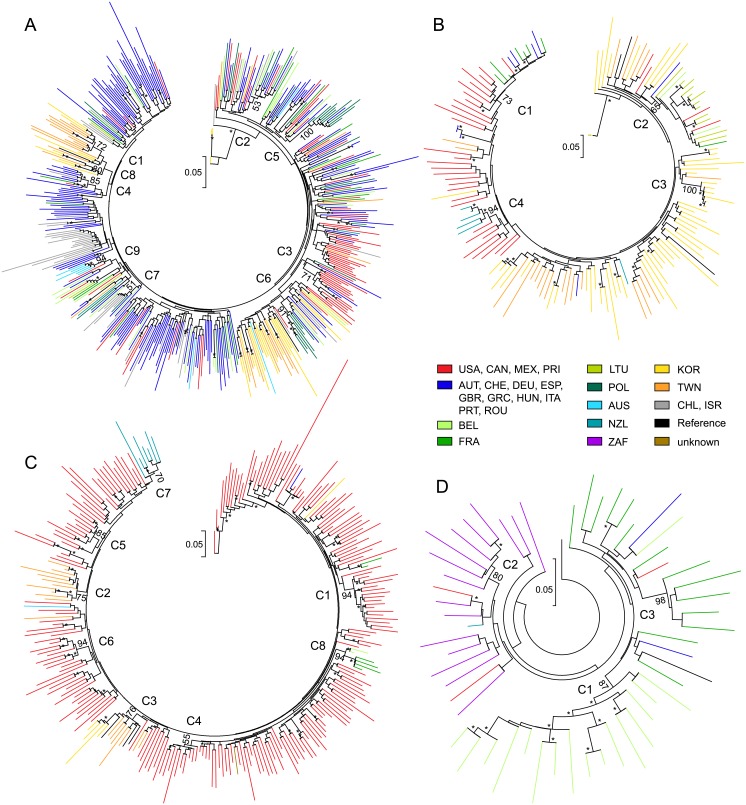
Phylogenetic clustering by geographic region in HCV subtypes 1b, 2a, 2b, and 5a. Maximum likelihood phylogenetic trees displayed in circular format for NS5A sequences from (A) GT1b-infected patients, (B) GT2a-infected patients, (C) GT2b-infected patients, and (D) GT5a-infected patients. Bootstrap values are listed for nodes of sequence clustering, and bootstrap values ≥70 at other nodes in the tree are marked with an asterisk (*). Sequence clusters by geographic region were numbered starting at C1. The genetic distance scale bar indicates the number of nucleotide substitutions per site between sequences. HCV patient isolates are represented by color based on the country of enrollment.

Phylogenetic analysis of 466 GT1b NS3/4A and NS5A sequences identified 9 sequence clusters with strong branch support, and clustering by country was most notable for sequences from Poland and Taiwan (Fig 3A). Cluster C1 (n = 13) contained sequences from Korea and Taiwan, and included 37% (11/30) of the total GT1b sequences from Taiwan. Among sequences from Poland, 45.7% (16/35) clustered in subgroups C5 and C6, which were comprised almost entirely (90–100%) of sequences from Poland (Table 1). Other smaller sequence clusters in subtype 1b included sequences exclusively from Asia (C8) or Australia (C9).

The prevalence of baseline polymorphisms in NS3 and NS5A was assessed by geographic region and phylogenetic cluster, and is shown in Tables 2 and 3, respectively. In GT1b, the prevalence of NS3 polymorphisms at amino acid positions 36, 54, 55, 155, and 168 was similar across geographic region and phylogenetic cluster (GT1b, Table 2). The most common polymorphism was Y56F, detected at 32% (147/461) prevalence overall. In general, Y56F was evenly distributed by geographic region, except that it was not detected in sequences from Oceania (0/10). The highest prevalence of Y56F was detected in sequences from Poland and Spain, with a prevalence of 51% (18/35) and 53% (18/34), respectively. Although the prevalence of Y56F was similar across geographic regions, the frequency of detection ranged from 0–100% in phylogenetic clusters C1-C9. Presence of Y56F was highest in clusters C7 (100%), C4 (70%) and C5 (60%), and detection was not limited to a specific country in those sequence clusters. NS3 Q80H/K/L/R polymorphisms were detected at an overall prevalence of 5% (23/461) in GT1b, but occurred at the highest prevalence in sequences from Asia (17.5%, 11/63). Polymorphisms at position Q80 were detected in 15–29% of sequences in clusters C1, C4, and C7.

In GT1b, the prevalence of polymorphisms in NS5A at amino acid positions 24, 28, 30, and 31 was similar across geographic region (GT1b, Table 3). Polymorphisms at positions P58, A92, and Y93 were detected at frequencies of 19% (12/63), 13% (8/63), and 13% (8/63), respectively, and were most prevalent in sequences from Asia compared to other geographic regions. Among the clusters containing sequences from Asia, cluster C1 contained 3 NS5A sequences with Y93H (3/12), and C8 contained 4 sequences with A92T (4/5) in NS5A.

Phylogenetic analysis of 152 GT2a sequences revealed that the majority of sequences from Europe (96.8%, 30/31) and some sequences from North America (32.4%, 11/34) clustered independently of sequences from Asia countries Korea and Taiwan (Fig 3B), and this difference was statistically significant (P-value <0.0001). Among the 4 sequence clusters identified by phylogenetic analysis, cluster C1 included sequences from Europe (58%) and North America (42%), cluster C2 included sequences from Europe (89%) and North America (11%), cluster C3 contained 7 sequences from Korea, and cluster C4 included 83% (5/6) of the total GT2a sequences from New Zealand (Table 1). Most of the sequences from France (7/8) were in cluster C1, while all (12/12) of the GT2a sequences from Lithuania sorted to cluster C2. Among 272 GT2b sequences, the majority of sequences from Europe (60%, 6/10), Asia (84%, 21/25), and New Zealand (100%, 7/7) clustered independently from North America (Fig 3C), and the difference was statistically significant (P-value <0.0001). Clusters C1, C4, C5, and C6 contained sequences exclusively from North America, while clusters C2 and C3 contained sequences from Korea and Taiwan, cluster C7 included 100% (7/7) of the GT2b sequences from New Zealand, and cluster C8 contained sequences from Europe (Table 1).

The most common NS3 polymorphism in GT2 sequences was Y56F, which occurred at a prevalence of 7% (10/144), 17% (44/254), 100% (71/71), and 65% (11/17) in subtypes 2a, 2b, 2c, and 2-other, respectively (Table 2). Among GT2a sequences from Asia (n = 75), 6 out of 7 sequences with Y56F in NS3 sorted in cluster C3, which was comprised entirely of sequences from Korea; similar clustering was not observed in other GT2 subtypes. In NS5A, specific clustering of baseline polymorphisms was not evident. Polymorphisms T24A/S were detected in 10% (15/150) of GT2a sequences, and 67% (4/6) of the sequences from New Zealand contained the T24A polymorphism and sorted in cluster C4 (Table 3). In NS5A, amino acid position 31 is polymorphic among GT2 sequences. The majority of GT2a and GT2-other sequences had M31, whereas most GT2b and GT2c sequences had L31 in NS5A. In GT2b-infected patients, the distribution of NS5A sequences with the common L/M31 polymorphisms varied by geographic region, and M31 occurred at a higher frequency in sequences from Asia (46%, 11/24) and North America (32%, 71/223), while L31 was most prevalent in sequences from Europe (89%, 8/9) and Oceania (100%, 8/8). In general, phylogenetic clustering in GT2b was differentiated by the presence or absence of 31L in NS5A.

In GT5a, phylogenetic analysis of 53 NS3/4A and NS5A sequences identified a large cluster of sequences from Belgium with strong branch support (Fig 3D). Cluster C1 (n = 16) contained sequences exclusively from Belgium, which included 94.1% (16/17) of the total GT5a sequences from Belgium. Two smaller clusters contained sequences from South Africa (n = 6) or France (n = 5). In NS3, D168E was the most common polymorphism, detected in 47% (25/53) of all GT5a sequences, and occurred at a similar frequency in all 3 sequence clusters (GT5a, Table 2). In NS5A, the Q30L/R polymorphism was only detected in sequences from Europe (3/33), while polymorphisms at positions L31, P58, and A92 were detected in sequences from South Africa (3/16; Table 3).

Sequence clustering by phylogenetic analysis was not detected in GT2c (n = 80; S1A Fig) or GT4d (n = 53; S1B Fig), and clustering in GT6a (n = 31) was minimal and included 1 cluster of 7 sequences (S1C Fig, Table 1). Minimal clustering was also detected in GT3a (n = 627), and included 1 cluster of 6 sequences (S2 Fig, Table 1). In NS3, Q168K/R was detected in 2% (10/624) of GT3a sequences. The F43L polymorphism in NS3 was detected in a single GT3a sequence; no other polymorphisms were detected at NS3 amino acid position 43. In NS5A, baseline polymorphisms A30L/M/R/S/T/V in GT3a were more prevalent in sequences from Oceania (8.2%, 13/159), while A30K prevalence was 2-fold higher in Europe (9.7%, 18/186) compared to North America (4.6%, 13/281) and Oceania (5.0%, 8/159). Y93H in GT3a was 2-fold more prevalent in Oceania than in North America or Europe (Table 3); one sequence from Europe had A30K as well as Y93H in NS5A.

### Two clades identified by phylogenetic analysis in HCV subtypes 4a and 6e

**Fig 4:**
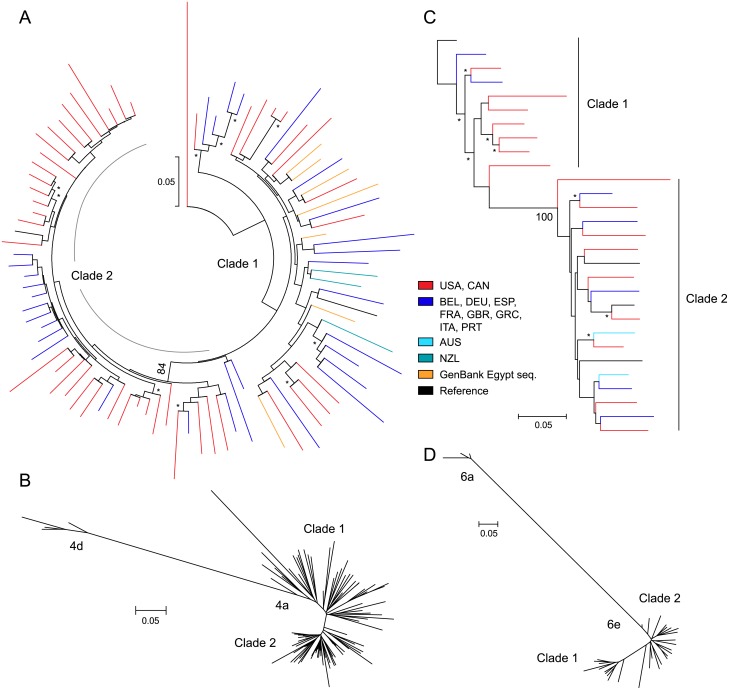
Two clades identified by phylogenetic analysis in HCV GT4a and GT6e. Maximum likelihood phylogenetic trees displayed for NS5A sequences from (A, B) GT4a-infected patients and (C, D) GT6e-infected patients. Bootstrap values ≥70 are marked with an asterisk (*), and values are listed at the nodes of divergence. The genetic distance scale bar indicates the number of nucleotide substitutions per site between sequences. HCV patient isolates are represented by color based on the country of enrollment.

Phylogenetic analysis of 82 HCV GT4a sequences identified 2 clades in subtype 4a (Fig 4A and 4B). The region and country distribution of clade 1 (n = 35) was 40% North America, 51% Europe, and 9% New Zealand. The distribution of clade 2 (n = 47) was 70% North America and 30% Europe (Table 1). The prevalence of sequences from North America was significantly higher in clade 2 (P-value = 0.008, two-tailed Fisher’s exact test), whereas the distribution of sequences from Europe was not significantly different between the 2 clades. The prevalence of baseline polymorphisms in NS3 and NS5A was assessed by geographic region and phylogenetic clade, and is shown in Tables 2 and 3, respectively. In NS3, T54S was detected at a prevalence of 5% (4/81) overall and was evenly distributed between the 2 phylogenetic clades (GT4a, Table 2). In NS5A, polymorphisms were detected at amino acid positions L28, L30, and P58 among 81 GT4a sequences (GT4a, Table 3). NS5A polymorphisms L28M and P58L/S/T were evenly distributed among the 2 clades. The L30R polymorphism was differentially distributed between clade 1 and clade 2, occurring at a frequency of 17% (6/35) in clade 1 versus 4% (2/46) in clade 2, although the difference did not reach statistical significance (P-value = 0.07, two-tailed Fisher’s exact test).

Two clades were also identified in GT6e (Fig 4C and 4D) by phylogenetic analysis of 25 NS3/4A and NS5A sequences. The geographic region distribution was not statistically different between the 2 clades with relatively small numbers of sequences. In GT6e, the distribution of clade 1 (n = 9) was 78% North America and 22% Europe, and clade 2 (n = 16) was 56% North America, 31% Europe, and 13% Australia (Table 1). In NS3, the only polymorphism detected was V36L at 8% (2/24) overall prevalence; V36L was detected exclusively in clade 2 at 12.5% (2/16) prevalence (GT6e, Table 2). NS5A baseline polymorphisms K24R, V28M, L31I, P58S, and T93S were detected in 52% (13/25) of GT6e sequences, and were each differentially distributed between clade 1 and clade 2 (GT6e, Table 3). K24R was detected in 56% (5/9) of sequences in clade 1, but was not detected in any (0/16) sequences in clade 2, and this difference was statistically significant (P-value = 0.002, two-tailed Fisher’s exact test). In addition, the distribution of V28M was significantly different between the 2 clades, with 89% (8/9) of sequences in clade 1 versus 12.5% (2/16) of sequences in clade 2 containing the V28M polymorphism (P-value = 0.0003, two-tailed Fisher’s exact test). The polymorphisms L31I, P58S, and T93S were only detected in sequences that sorted to clade 2.

### Activity of glecaprevir or pibrentasvir against HCV replicons containing NS3/4A or NS5A from GT1-6 clinical samples

Antiviral activity of GLE was assessed against a panel of 49 NS3/4A GT1-6 clinical samples, which included 2 GT1, 5 GT2, 1 GT3, 7 GT4, 1 GT5, and 1 GT6 subtypes (Table 4). GLE retained activity against all NS3/4A isolates tested in the transient replicon assay. The GLE median EC_50_ for subtypes 1a, 1b, and 5a was 0.08, 0.29, and 0.12 nM, respectively [34]. In GT2, GLE had a median EC_50_ value of 1.6, 2.2, 0.50, 1.7, and 5.1 nM against subtypes 2a, 2b, 2c, 2i, and 2l, respectively. In GT3a, one sample with A166 had a mean EC_50_ of 3.8 nM, and one sample with A166S had an EC_50_ of 0.71 nM. Among 7 GT4 subtypes, the GLE median EC_50_ ranged from 0.08 nM to 0.91 nM. GLE was also active against a clone of GT6e with an EC_50_ of 0.09 nM. The majority of NS3/4A clinical samples did not have polymorphisms at NS3 amino acid positions 36, 43, 54, 55, 56, 80, 155, 156, or 168.

**Table 4:** Activity of glecaprevir or pibrentasvir against a panel of transient replicons containing NS3 or NS5A, respectively, from HCV GT1-6 clinical samples.

HCV Subtype	NS3 Amino Acid Polymorphisms^a^	N^b^	Glecaprevir Median EC_50_ [Reference], nM	NS5A Amino Acid Polymorphisms^a^	N^b^	Pibrentasvir Median EC_50_ (Range), pM
1a	None	5	0.06 [34]^c^	None	11	0.89 (0.55–1.7)^d^
	Q80K/N	6	0.09 [34]^c^			
1b	None	9	0.29 [34]^c^	None	7	2.9 (2.1–3.5)
				L31M	1	1.4
2a	None	4	1.6 [34]^c^	None	1	1.9
				L31M	5	0.91 (0.52–1.1)
2b	None	4	2.2 [34]^c^	None	2	1.4 (1.3–1.5)
				M31L	7	1.3 (1.1–1.5)
				M31L/M	2	1.6 (1.3–1.9)
2c	None	1	0.50	None	1	1.8
				R30K	2	3.5 (2.5–4.5)
				F28C+R30K	1	2.7
				R30K/R, P58P/A	1	1.4
				S24A/S, F28C+R30K	1	5.8
				F28C/F, R30K+L31F	1	10.2
2i	None	1	1.7	None	2	0.67 (0.61–0.73)
				K30K/R	1	0.73
2l	None	1	5.1	S92C	1	0.83
3a	None	1	3.8	None	8	0.66 (0.47–1.2)
	A166S	1	0.71	A30K	3	0.79 (0.61–1.1)
				A30T	1	0.74
				Y93H	2	1.4 (1.1–1.7)
3b	ND^e^			V31M	1	15.6
4a	None	5	0.39 [34]^c^	None	4	0.50 (0.45–0.59)
	T54S	1	0.44 [34]^c^	K24R	1	0.57
				L28M	1	0.44
				L30R	1	1.3
				P58T	1	0.27
4b	ND^e^			T92A	1	1.2
				S30L+P58S+T92A	1	1.8
				S30L+P58T+T92A+H93Y	1	0.45
4d	None	3	0.17 [34]^c^	None	2	1.3 (1.1–1.5)
				T58L	1	0.98
				T58P	2	1.5 (1.1–1.8)
				T58S	1	1.5
				M31V+T58P	1	1.4
4f	None	1	0.12	Q30R	4	2.3 (0.97–10.0)
				Q30R+M31L	1	10.3
4g	None	1	0.08	L30C+M31L+ H93Y	2	0.67 (0.59–0.75)
				L28M+L30R+ M31L+P58S+ H93Y	1	1.6
4k	None	1	0.24	None	1	1.1
				M31L	1	1.1
4o	None	1	0.16	None	4	0.91 (0.72–1.1)
				T30A/V	1	0.43
4r	None	1	0.91	None	2	1.3 (1.2–1.3)
				I28V+R30H	1	0.71
				I28V, Y93H/Y	1	0.96
				I28M+L31M+ P58A	1	0.49
5a	None	1	0.12 [34]^c^	None	1	1.1^d^
6a	ND^e^			None	2	0.49 (0.34–0.63)
				F28L	2	0.89 (0.74–1.0)
6e	None	1	0.09	K24R+V28M	1	0.83^d^
				P58P/S, T93T/S	1	1.2
6p	ND^e^			None	1	0.50^d^

^a^Polymorphisms are listed at signature amino acid positions in NS3 and NS5A. None indicates that there were no polymorphisms relative to the subtype-specific reference sequence listed in S2 or S3 Tables at any of the specified amino acid positions. HCV patient samples for NS3 and NS5A are distinct and data in each row does not correspond to the same patient sample.

^b^N, number of patient samples

^c^Glecaprevir median EC_50_ value and range was published in Ref. [34].

^d^Pibrentasvir median EC_50_ value and range was published in Ref. [35].

^e^ND, EC_50_ was not determined

Antiviral activity of PIB was assessed against a panel of 108 NS5A GT1-6 clinical samples that included 2 GT1, 5 GT2, 2 GT3, 8 GT4, 1 GT5, and 3 GT6 subtypes (Table 4). PIB retained activity against the majority of NS5A clinical samples tested in the HCV transient replicon assay, including isolates which contained polymorphisms in NS5A at positions important for the inhibitor class (Table 4). The PIB median EC_50_ was 0.89, 2.7, and 1.1 pM against subtypes 1a, 1b, and 5a, respectively [35]. In GT2, PIB had a median EC_50_ of 0.93, 1.3, 2.7, 0.73, and 0.83 pM against subtypes 2a, 2b, 2c, 2i, and 2l, respectively. Among 7 GT2c samples analyzed in the transient replicon assay, PIB had reduced activity against one GT2c sample with F28C/F and R30K+L31F in NS5A (EC_50_ = 10.2 pM); this patient achieved SVR12 on a regimen of GLE/PIB for 12 weeks duration. NS5A polymorphisms F28C, R30K, and L31F were detected at a frequency of 43% (34/80), 90% (72/80), and 3.8% (3/80), respectively, among GT2c-infected patients (Table 3). In GT3a, the median EC_50_ for PIB was 0.66 pM for clinical samples with no NS5A polymorphisms, 0.79 pM with A30K in NS5A, and 1.4 pM with Y93H in NS5A. In GT3b, PIB had a reduction in activity against a replicon containing a GT3b NS5A consensus sequence with K30 and V31M in NS5A, with an EC_50_ of 15.6 pM. Compared to GT3a sequences where A30 and L31 were detected as the predominant amino acids in NS5A (Table 3), amino acids K30 and M31 were the most prevalent in GT3b sequences (n = 6). A recent study compared the activity of PIB and other NS5A inhibitors against chimeric HCV replicons containing NS5A from either GT3a or GT3b, and attributed the differences in NS5A inhibitor activity between subtypes 3a and 3b to the amino acid differences at positions 30 and/or 31 in NS5A [39]. Among 8 GT4 subtypes, the PIB median EC_50_ ranged from 0.50 pM to 2.9 pM. Two GT4f samples with Q30R or Q30R+L31M in NS5A had reduced susceptibility to PIB (EC_50_ = 10 pM); both patients were enrolled in a clinical study evaluating the regimen of ombitasvir/paritaprevir/ritonavir with or without RBV for HCV GT4 infection, and both patients achieved SVR12 with this regimen. PIB retained activity against subtypes 6a, 6e, and 6p, with median EC_50_ values of 0.69, 1.0, and 0.50 pM, respectively.

## Discussion

In this report, a large dataset of HCV GT1-6 NS3/4A and NS5A sequences was utilized to assess genetic diversity within HCV subtypes by geographic region. Among NS3/4A and NS5A sequences isolated from 2348 patient samples, phylogenetic analysis identified 6 genotypes and 44 subtypes, including 3 GT1, 8 GT2, 3 GT3, 13 GT4, 1 GT5, and 16 GT6 subtypes (Fig 1). In addition, we analyzed 20 GT2 samples where the subtype could not be determined by phylogenetic analysis due to lack of homology with the 11 confirmed GT2 subtypes, potentially representing novel GT2 subtypes. Subtype diversity was highest in GT2, GT4, and GT6, which are reported to be the most diverse genotypes and encompass 52 confirmed subtypes [5]. We detected phylogenetic clustering by country in HCV subtypes 1a, 1b, 2a, 2b, and 5a, suggesting that genetically distinct virus lineages are circulating in different countries. Since the prevalence of NS3 and NS5A baseline polymorphisms varied substantially by genotype and subtype among patients treated with a regimen of GLE/PIB, we also determined the activity of GLE or PIB against replicons containing NS3/4A or NS5A from HCV GT1-6 clinical samples representing 6 genotypes and 21 subtypes overall. In the transient HCV replicon assay, GLE and PIB retained activity against the majority of HCV replicons containing NS3/4A or NS5A from HCV GT1-6 clinical samples, confirming previous reports describing the pangenotypic activity of GLE and PIB [34, 35]. A separate publication [39] recently presented the pooled resistance analysis of HCV GT1-6 infected patients treated with GLE/PIB in 8 registrational clinical studies, and revealed a lack of impact of genotype, subtype, or baseline polymorphism prevalence on treatment outcome with the recommended treatment duration [39, 48, 49].

HCV GT1 infection is the most prevalent and geographically disseminated genotype globally [6]. HCV subtype 1a is more common in North America, Andean Latin America, and Australia [6, 50], and consists of two distinct phylogenetic clades [9, 10]. Consistent with previous studies, our phylogenetic analysis of 395 HCV GT1a sequences from 22 countries confirmed the presence of 2 clades in subtype 1a, which differed by geographic region and NS3 Q80K prevalence. Similar to published reports [9, 51], we found that the distribution of sequences from North America and the prevalence of the GT1a NS3 Q80K polymorphism were significantly higher in clade 1, while sequences from Europe were more common in clade 2 (P-value <0.0001). In our analysis, Q80K prevalence was significantly higher in sequences from North America (56%) compared to Europe (21%) or Oceania (2.5%; P-value <0.0001). The origin for this difference has been traced to a single virus lineage with Q80K in NS3 that occurred in the United States around 1940 [52].

Sub-clustering detected in subtype 1a, clade 1 in our analysis was generally grouped by the presence or absence of Q80K in NS3. 94.7% of Australian sequences in clade 1 contained Q80 in NS3 and clustered in a strongly supported sub-cluster with other NS3 Q80 sequences, likely representing previously described sub-clade 1C [51]. Phylogenetic separation has been reported for subtype 1a sequences from North America and Australia [53], and Bayesian estimates place the origin of the epidemics for both continents around the early 20^th^ century coinciding with World War I [54]. Geographical separation of the continents likely resulted in genetically distinct sequences due to a founder effect in the two regions, which may also explain the relative absence of the NS3 Q80K polymorphism in the Australian sequences. While NS3 sequences have been analyzed extensively in subtype 1a due to the impact of Q80K on SVR rates with regimens containing some HCV protease inhibitors [55], corresponding data for NS5A has not been widely reported. Our analysis of NS5A sequences in subtype 1a revealed identical phylogenetic separation by clade, and NS5A polymorphisms at positions important for the inhibitor-class had a similar prevalence between clade 1 and 2.

Phylogenetic clustering by geographic region has been reported for some HCV subtypes [8].

We detected phylogenetic clustering by country in HCV subtypes 1b, 2a, 2b, and 5a, likely due to geographical separation resulting in genetically distinct virus lineages. Among 466 GT1b sequences from 24 countries included in our analysis, clustering by country was most notable for sequences from Poland and Taiwan (Fig 3A), where GT1b comprises 77% [56, 57] and 46% [58] of HCV infections, respectively. Incidence of HCV infection in Poland and Taiwan is higher in the intravenous drug use (IDU) population [59, 60], and phylogenetic clustering has been reported for networks of people who inject drugs [61]. However, only 1 out of 65 total patients from Poland or Taiwan reported a history of IDU in our GT1b-infected patient population, so clustering of sequences from Poland and Taiwan is likely due to geographical separation rather than independent networks of IDU individuals.

Our analysis of HCV subtypes 2a and 2b detected independent clustering for the majority of sequences from Europe, New Zealand, Asia, and North America (Fig 3B and 3C). In GT2b-infected patients, the distribution of NS5A sequences with L/M31 polymorphisms varied by geographic region and phylogenetic cluster. Due to epidemiological differences among GT2 subtypes and a more limited global distribution for GT2 in general [1], clustering by country in GT2a and GT2b is likely due to the geographical separation of Europe, New Zealand, Asia, and North America. Similarly, in our analysis of 53 GT5a sequences, three phylogenetic clusters were detected representing the countries of Belgium, France, and South Africa (Fig 3D). GT5a is the most prevalent genotype in South Africa [6, 57], but high prevalence of GT5a has also been reported in limited geographic areas of France [62, 63] and Belgium [64]. Phylogenetic clustering has been reported for GT5a sequences originating from Belgium [64], and Bayesian phylogeny estimated the time to most recent common ancestor for Belgian and South African isolates to the late 1800s, demonstrating independent populations of GT5a circulating for over 100 years in both Belgium and South Africa [65].

Our analyses of GT2c, GT3a, GT4d, and GT6a sequences did not detect phylogenetic clustering by geographic region or country (S1 and S2 Figs). Phylogenetic clustering by country has been reported for GT3a sequences from Pakistan [66], India [8], and Russia [8], where GT3a occurs at a prevalence of 79% [67], 64% [57], and 36% [57], respectively, among HCV-infected individuals. While GT3a is the most prevalent genotype in Pakistan and India, our clinical studies did not enroll patients from these countries, likely explaining why we failed to see significant clustering in GT3a.

Two clades were detected in GT4a by phylogenetic analysis in our study, and we found that the prevalence of sequences from North America was higher in clade 2 based on country of enrollment. HCV GT4 infection occurs at a low frequency in HCV-infected patients from North America, and is most prevalent in Central and Eastern sub-Saharan Africa, North Africa, and the Middle East [1, 6, 68]. Egypt has the highest prevalence of GT4 infection worldwide [68, 69], predominantly subtype 4a which spread rapidly due to anti-schistosomiasis campaigns beginning in the 1940s [69–71]. In a previous study that examined NS5A genetic diversity in HCV GT4-infected patients treated with OBV/PTV/r, subtype 4a sequences from Europe and the United States clustered independently from 4a sequences from Egypt in a phylogenetic analysis, and the L30R/S polymorphism in NS5A was significantly associated with the Egyptian cluster [40]. Since we did not collect country of origin information from GT4a-infected patients in our clinical studies, we included 7 GT4a NS3/4A and NS5A sequences from GenBank that were identified as originating from Egypt in our phylogenetic analyses (Fig 4A) and found that the Egyptian GT4a sequences all sorted to clade 1. NS5A baseline polymorphism analysis also revealed that prevalence of L30R was numerically higher in clade 1 (17%) versus clade 2 (4%). Based on these combined observations, we propose that subtype 4a clade 1 may be associated with sequences from Egypt and characterized by the presence of the NS5A L30R polymorphism; this hypothesis should be investigated in future studies.

In GT6e, two clades were also identified by phylogenetic analysis of 25 NS3/4A and NS5A sequences (Fig 4C and 4D), and NS5A amino acid polymorphisms were differentially distributed between the 2 clades. Based on country of enrollment, the geographic region distribution was not statistically different between the 2 clades. However, most of the GT6e-infected patients were enrolled from North America and Europe where GT6 infection is relatively rare, and the patients’ country of origin is not known. HCV GT6 infection is most prevalent in East and Southeast Asia [1, 72], and outside of those regions GT6 infection is generally found in emigrant populations from endemic countries [72, 73]. Subtype 6e occurs at a high frequency in Vietnam where GT6 comprises around 54% of HCV infections [74, 75]. Limited data have been published for subtype 6e describing genetic diversity and prevalence of baseline polymorphisms in NS3 and NS5A [8]. Among 25 GT6e-infected patients included in our analysis, V36L in NS3 was detected in 8% (2/24) of sequences, and was exclusively detected in clade 2. NS5A baseline polymorphisms K24R, V28M, L31I, P58S, and T93S were detected in 52% (13/25) of GT6e sequences, and were each differentially distributed between clade 1 and clade 2 (Table 3). NS5A polymorphism K24R was exclusively detected in clade 1, while L31I, P58S, and T93S were only detected in clade 2, and V28M prevalence was significantly higher in clade 1 (89%) versus clade 2 (12%). Our analysis provides additional data illustrating the genetic diversity of subtype 6e.

In conclusion, this study examined HCV genetic diversity among 6 genotypes and 44 subtypes identified from 2348 HCV-infected patients treated with a regimen of GLE/PIB who were enrolled in 27 countries, thus expanding the available data on HCV epidemiology, subtype diversity, and NS3/NS5A baseline polymorphism prevalence at amino acid positions important for the inhibitor class. The efficacy of DAA regimens for the treatment of HCV infection can vary by HCV genotype, subtype, and the presence of baseline polymorphisms, although newly approved pangenotypic DAA regimens are less impacted by these variables [11, 23, 39]. While phylogenetic clustering by country is still detected for some HCV subtypes, representing genetically distinct virus lineages circulating in specific countries or regions, the global distribution of subtypes and intra-subtype virus lineages appears to be shifting with increased immigration patterns. The availability of DAA regimens for HCV treatment varies by country worldwide [74], and continued effort is required to ensure pangenotypic DAA regimens are available for all HCV-infected patients in order to achieve HCV elimination worldwide.

## Supporting information

S1 Table Country of enrollment by ISO country code. (DOCX)

S2 Table HCV subtype reference sequences for NS3/4A. (DOCX)

S3 Table HCV subtype reference sequences for NS5A. (DOCX)

S1 Fig Phylogenetic analysis of HCV subtypes 2c, 4d, and 6a. Maximum likelihood phylogenetic trees displayed for NS3/4A sequences from (A) GT2c-infected patients, (B) GT4d-infected patients, and (C) GT6a-infected patients. Bootstrap values are listed for nodes of sequence clustering, and bootstrap values ≥70 at other nodes in the tree are marked with an asterisk (*). Sequence clusters by geographic region were numbered starting at C1. The genetic distance scale bar indicates the number of nucleotide substitutions per site between sequences. HCV patient isolates are represented by color based on the country of enrollment.
(TIF)

S2 Fig Phylogenetic analysis of HCV GT3a sequences. Maximum likelihood phylogenetic tree displayed in circular format for NS5A sequences from GT3a-infected patients. Bootstrap values are listed for nodes of sequence clustering, and bootstrap values ≥70 at other nodes in the tree are marked with an asterisk (*). The genetic distance scale bar indicates the number of nucleotide substitutions per site between sequences.
(TIF)
