# Supplementary material for: Hepatitis C virus genetic diversity by geographic region within genotype 1-6 subtypes among patients treated with glecaprevir and pibrentasvir
Source: PLoS One. 2018 Oct 4;13(10):e0205186. doi: 10.1371/journal.pone.0205186 (PMC6171933; doi:10.1371/journal.pone.0205186)
Supplement: S1 Table — (DOCX) [file pone.0205186.s001.docx]

**S1 Table. Country of enrollment by ISO country code.**

| **ISO Country Code** | **Country** | **Geographic Region** |
| --- | --- | --- |
| AUS | Australia | Oceania |
| AUT | Austria | Europe |
| BEL | Belgium | Europe |
| CAN | Canada | North America |
| CHE | Switzerland | Europe |
| CHL | Chile | Rest-of-world |
| DEU | Germany | Europe |
| ESP | Spain | Europe |
| FRA | France | Europe |
| GBR | United Kingdom | Europe |
| GRC | Greece | Europe |
| HUN | Hungary | Europe |
| ISR | Israel | Rest-of-world |
| ITA | Italy | Europe |
| KOR | Korea (South) | Asia |
| LTU | Lithuania | Europe |
| MEX | Mexico | North America |
| NZL | New Zealand | Oceania |
| POL | Poland | Europe |
| PRI | Puerto Rico | North America |
| PRT | Portugal | Europe |
| ROU | Romania | Europe |
| SGP | Singapore | Asia |
| SWE | Sweden | Europe |
| TWN | Taiwan | Asia |
| USA | United States of America | North America |
| ZAF | South Africa | Rest-of-world |
