# Supplementary material for: Hepatitis C virus genetic diversity by geographic region within genotype 1-6 subtypes among patients treated with glecaprevir and pibrentasvir
Source: PLoS One. 2018 Oct 4;13(10):e0205186. doi: 10.1371/journal.pone.0205186 (PMC6171933; doi:10.1371/journal.pone.0205186)
Supplement: S2 Table — (DOCX) [file pone.0205186.s002.docx]

**S2 Table. HCV subtype reference sequences for NS3/4A.**

| **Genotype** | **GenBank Accession Number** | **Strain** | **NS3 Amino Acid Position** | | | | | | | | |
| --- | --- | --- | --- | --- | --- | --- | --- | --- | --- | --- | --- |
|  |  |  | **36** | **43** | **54** | **55** | **56** | **80** | **155** | **156** | **168** |
| 1a | NC_004102 | H77 | V | F | T | V | Y | Q | R | A | D |
| 1b | AJ238799 | Con1 | V | F | T | V | Y | Q | R | A | D |
| 2a | AB047639 | JFH-1 | L | F | T | V | Y | G | R | A | D |
| 2b | D10988 | HC-J8 | L | F | T | V | Y | G | R | A | D |
| 2c | D50409 | BEBE1 | L | F | T | V | F | G | R | A | D |
| 2i | DQ155561 | D54 | L | F | T | V | F | G | R | A | D |
| 2l | KC197235 | MRS89 | L | F | T | V | Y | G | R | A | D |
| 3a^a^ | GU814263 | S52 | L | F | T | V | Y | Q | R | A | Q |
| 4a | GU814265 | ED43 | L | F | T | V | Y | Q | R | A | D |
| 4d | FJ462437 | QC382 | L | F | T | V | Y | Q | R | A | D |
| 4f | EF589161 | IFBT88 | L | F | T | V | Y | Q | R | A | D |
| 4g | FJ462432 | QC193 | L | F | T | V | Y | Q | R | A | D |
| 4k | FJ462438 | QC383 | L | F | T | V | Y | Q | R | A | D |
| 4o | FJ462440 | QC93 | L | F | T | V | Y | Q | R | A | D |
| 4r | FJ462439 | QC384 | L | F | T | V | Y | Q | R | A | D |
| 5a | AF064490 | SA13 | L | F | T | V | F | K | R | A | D |
| 6a | Y12083 | EUHK2 | V | F | T | V | Y | L | R | A | D |
| 6e | DQ314805 | GX004 | V | F | T | V | Y | Q | R | A | D |

a. Amino acid position 166 is A in GT3a.
