# Supplementary material for: Hepatitis C virus genetic diversity by geographic region within genotype 1-6 subtypes among patients treated with glecaprevir and pibrentasvir
Source: PLoS One. 2018 Oct 4;13(10):e0205186. doi: 10.1371/journal.pone.0205186 (PMC6171933; doi:10.1371/journal.pone.0205186)
Supplement: S3 Table — (DOCX) [file pone.0205186.s003.docx]

**S3 Table. HCV subtype reference sequences for NS5A.**

| **Genotype** | **GenBank Accession Number** | **Strain** | **NS5A Amino Acid Position** | | | | | | | |
| --- | --- | --- | --- | --- | --- | --- | --- | --- | --- | --- |
|  |  |  | **24** | **28** | **30** | **31** | **32** | **58** | **92** | **93** |
| 1a | NC_004102 | H77 | K | M | Q | L | P | H | A | Y |
| 1b | AJ238799 | Con1 | Q | L | R | L | P | P | A | Y |
| 2a | AB047639 | JFH-1 | T | F | K | L | P | P | C | Y |
| 2b | D10988 | HC-J8 | S | L | K | M | P | P | C | Y |
| 2c | D50409 | BEBE1 | S | F | R | L | P | P | C | Y |
| 2i | DQ155561 | D54 | S | F | K | M | P | P | C | Y |
| 2l | KC197235 | MRS89 | S | L | K | L | P | P | S | Y |
| 3a | GU814263 | S52 | S | M | A | L | P | P | E | Y |
| 3b | D49374 | HCV-Tr | S | M | K | V | P | P | E | Y |
| 4a | GU814265 | ED43 | K | L | L | M | P | P | A | Y |
| 4b | FJ462435 | QC264 | K | L | S | M | P | P | T | H |
| 4d | FJ462437 | QC382 | K | L | R | M | P | T | A | Y |
| 4f | EF589161 | IFBT88 | K | L | Q | M | P | P | A | Y |
| 4g | FJ462432 | QC193 | K | L | L | M | P | P | A | H |
| 4k | FJ462438 | QC383 | K | L | R | M | P | P | A | Y |
| 4o | FJ462440 | QC93 | K | M | T | M | P | P | A | Y |
| 4r | FJ462439 | QC384 | K | I | R | L | P | P | A | Y |
| 5a | AF064490 | SA13 | Q | L | Q | L | P | P | A | T |
| 6a | Y12083 | EUHK2 | Q | F | R | L | P | T | A | T |
| 6e | DQ314805 | GX004 | K | V | S | L | P | P | A | T |
| 6p | EF424626 | QC216 | K | V | S | L | P | P | A | T |
