# Supplementary figures and images for: Hepatitis C virus genetic diversity by geographic region within genotype 1-6 subtypes among patients treated with glecaprevir and pibrentasvir
Source: PLoS One. 2018 Oct 4;13(10):e0205186. doi: 10.1371/journal.pone.0205186 (PMC6171933; doi:10.1371/journal.pone.0205186)

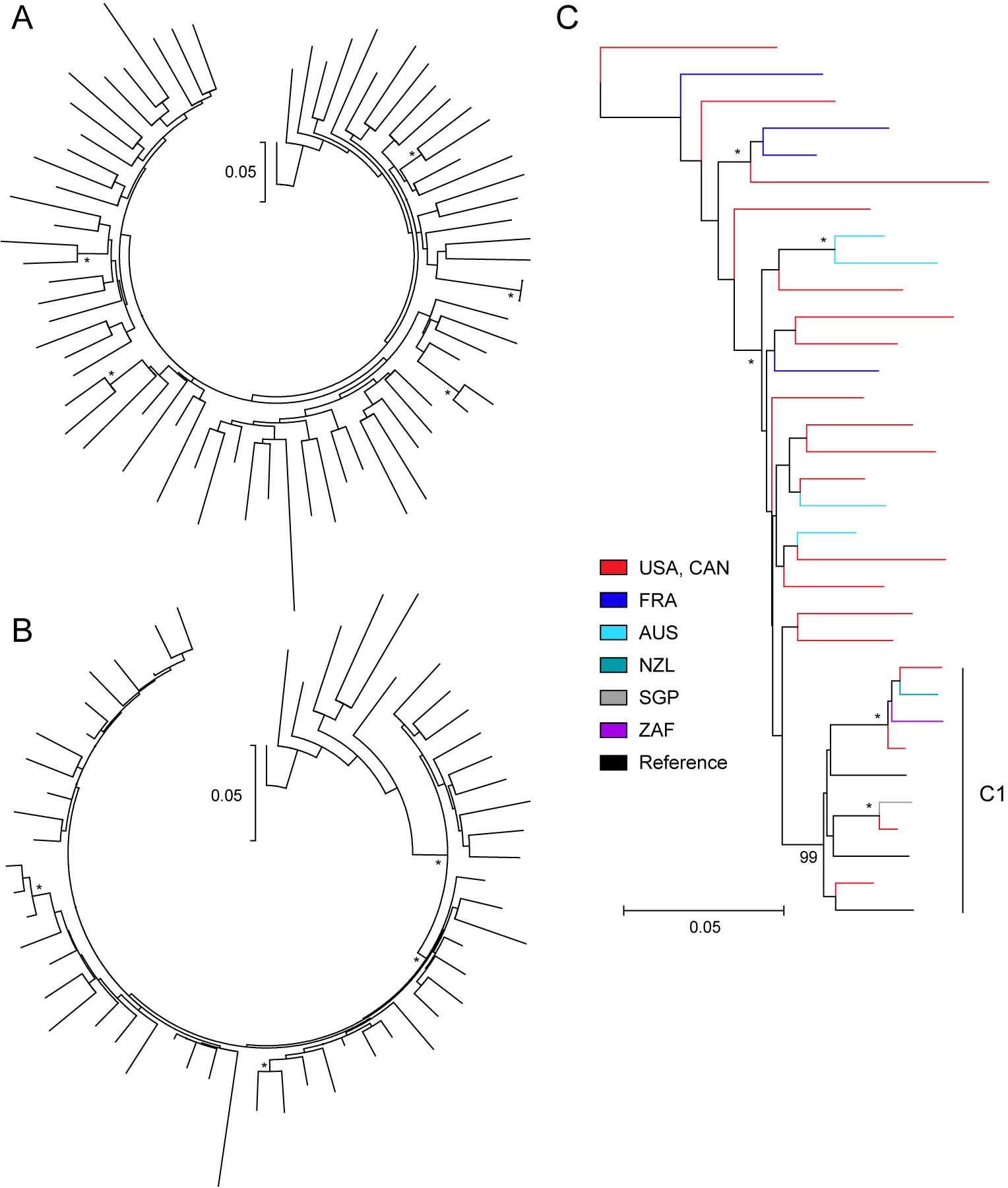

Supplement: S1 Fig — Maximum likelihood phylogenetic trees displayed for NS3/4A sequences from (A) GT2c-infected patients, (B) GT4d-infected patients, and (C) GT6a-infected patients. Bootstrap values are listed for nodes of sequence clustering, and bootstrap values ≥70 at other nodes in the tree are marked with an asterisk (*). Sequence clusters by geographic region were numbered starting at C1. The genetic distance scale bar indicates the number of nucleotide substitutions per site between sequences. HCV patient isolates are represented by color based on the country of enrollment. (TIF) [file pone.0205186.s004.tif]

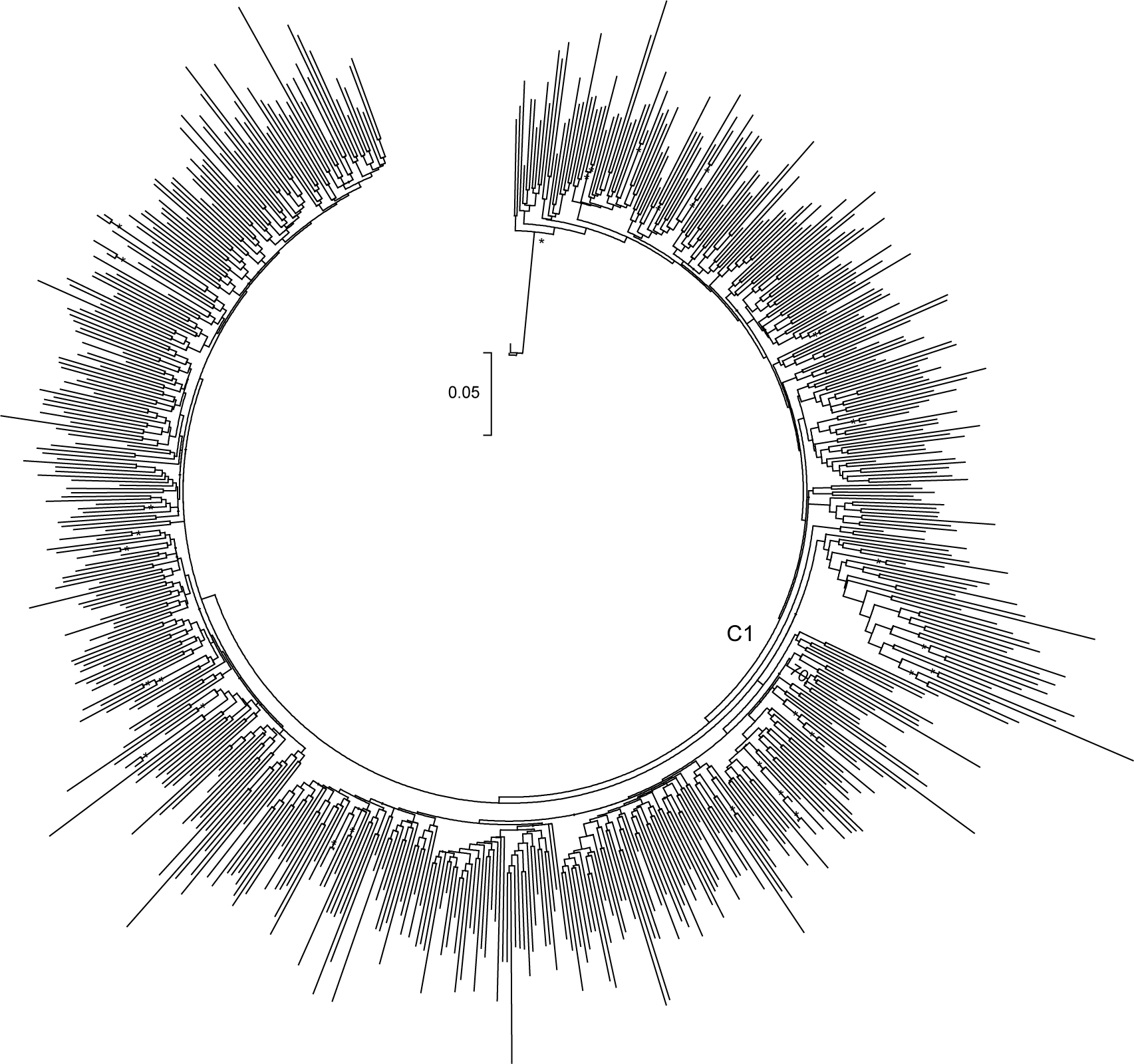

Supplement: S2 Fig — Maximum likelihood phylogenetic tree displayed in circular format for NS5A sequences from GT3a-infected patients. Bootstrap values are listed for nodes of sequence clustering, and bootstrap values ≥70 at other nodes in the tree are marked with an asterisk (*). The genetic distance scale bar indicates the number of nucleotide substitutions per site between sequences. (TIF) [file pone.0205186.s005.tif]
